# Roquin1 inhibits the proliferation of breast cancer cells by inducing G1/S cell cycle arrest via selectively destabilizing the mRNAs of cell cycle–promoting genes

**DOI:** 10.1186/s13046-020-01766-w

**Published:** 2020-11-23

**Authors:** Wenbao Lu, Meicen Zhou, Bing Wang, Xueting Liu, Bingwei Li

**Affiliations:** 1 Institute of Microcirculation, Chinese Academy of Medical Sciences & Peking Union Medical College, #69 Dongdan Beidajie, DongCheng District, Beijing, 100005 China; 2 Department of Endocrinology, Beijing Jishuitan Hospatial, The 4th Clinical Medical College of Peking University, Beijing, 100035 China

**Keywords:** Roquin1, Breast cancer, Cell cycle, Cyclin E1, MCM2, mRNA decay

## Abstract

**Background:**

Dysregulation of cell cycle progression is a common feature of human cancer cells; however, its mechanism remains unclear. This study aims to clarify the role and the underlying mechanisms of Roquin1 in cell cycle arrest in breast cancer.

**Methods:**

Public cancer databases were analyzed to identify the expression pattern of Roquin1 in human breast cancers and its association with patient survival. Quantitative real-time PCR and Western blots were performed to detect the expression of Roquin1 in breast cancer samples and cell lines. Cell counting, MTT assays, flow cytometry, and in vivo analyses were conducted to investigate the effects of Roquin1 on cell proliferation, cell cycle progression and tumor progression. RNA sequencing was applied to identify the differentially expressed genes regulated by Roquin1. RNA immunoprecipitation assay, luciferase reporter assay, mRNA half-life detection, RNA affinity binding assay, and RIP-ChIP were used to explore the molecular mechanisms of Roquin1.

**Results:**

We showed that Roquin1 expression in breast cancer tissues and cell lines was inhibited, and the reduction in Roquin1 expression was associated with poor overall survival and relapse-free survival of patients with breast cancer. Roquin1 overexpression inhibited cell proliferation and induced G1/S cell cycle arrest without causing significant apoptosis. In contrast, knockdown of Roquin1 promoted cell growth and cycle progression. Moreover, in vivo induction of Roquin1 by adenovirus significantly suppressed breast tumor growth and metastasis. Mechanistically, Roquin1 selectively destabilizes cell cycle–promoting genes, including Cyclin D1, Cyclin E1, cyclin dependent kinase 6 (CDK6) and minichromosome maintenance 2 (MCM2), by targeting the stem–loop structure in the 3′ untranslated region (3’UTR) of mRNAs via its ROQ domain, leading to the downregulation of cell cycle–promoting mRNAs.

**Conclusions:**

Our findings demonstrated that Roquin1 is a novel breast tumor suppressor and could induce G1/S cell cycle arrest by selectively downregulating the expression of cell cycle–promoting genes, which might be a potential molecular target for breast cancer treatment.

**Supplementary Information:**

The online version contains supplementary material available at 10.1186/s13046-020-01766-w.

## Background

Breast cancer is the most common cancer among women worldwide, and the incidence rates of breast cancer have increased rapidly in China in recent years [1, 2]. Although the death rates of patients have decreased due to the early detection and advanced treatment in recent years, the complex mechanism of tumorigenesis and progression still impede the treatment of breast cancer. Therefore, further elucidation the molecular mechanisms underlying tumorigenesis and progression of breast cancer is still necessary.

Cell cycle dysregulation is a common feature of human cancers, including breast cancer, and is characterized by uncontrolled cell proliferation and cell cycle progression of cancer cells [3–5], which is one reason why tumor cells are capable of unlimited proliferation and resistance to conventional treatments. Although increasing studies have expanded the knowledge on cell cycle regulation, the post-transcriptional mechanism of cell cycle dysregulation in cancer cells, especially through RNA-binding proteins (RBPs), remains unclear.

Roquin1, a Cys-Cys-Cys-His-type RBP encoded by *Rc3h1*, was initially found to play an important role in immune regulation through its ubiquitin ligase activity [6, 7]. Roquin1 is rapidly induced in T cells upon stimulation by the inflammatory inhibitory factor interleukin-10 [8]. Furthermore, Roquin1 can suppress autoimmunity by destabilizing the inducible T cell costimulator (ICOS) mRNA through its ROQ domain [9]. Therefore, Roquin1 is regarded as a regulator of the immune system and orchestrates the differentiation of various immune cells, including follicular helper T cells (TfHs), Natural killer T (NkT), and regulatory T (Treg) cells [10–12]. Roquin1 deficiency induced the death of mouse embryos and severe autoimmune reactions and enteritis [13, 14]. Evidence has shown that Roquin1 can induce mRNA decay by binding the stem–loop structure in the 3’UTR of target genes [15–17]. In addition, Roquin1 serves as a regulator of multiple signaling pathways, such as AMP-activated protein kinase (AMPK) [18], NF-κB [19], and PI3K-mTOR [20], to regulate immune responses. Roquin1 is additionally able to regulate microRNA homeostasis [21]. In cancerous TfH cells, the *Roquin1* expression level was similar to that in normal TfH cells [22]. However, it remains unknown whether Roquin1 plays a role in cancer progression.

In this study, we showed that Roquin1 is a potent breast tumor suppressor that induces tumor cell cycle arrest by selectively suppressing the expression of cell cycle–promoting genes, including *CCND1*, *CCNE1*, *CDK6*, and *MCM2*. Roquin1 expression was reduced in breast cancer tissues and cells, which might contribute to their lack of cell cycle regulation. Ectopic Roquin1 expression induces G1/S cell cycle arrest in breast tumor cells. In contrast, further suppression of Roquin1 expression by shRNAs facilitated tumor cell proliferation and cell cycle progression. Consistent with these in vitro observations, Roquin1 expression in vivo significantly inhibited tumor growth and metastasis. By analyzing a database of human breast tumors [23], we found that low Roquin1 levels in tumor samples were strongly associated with poor survival of luminal A, luminal B, and basal breast cancer patients. Moreover, Roquin1 expression was negatively correlated with *CCNE1* and *MCM2* in human breast tumors. These results suggested that Roquin1 is a potential tumor suppressor that is involved in regulating cell cycle progression by suppressing cell cycle–promoting genes expression.

## Methods

### Animal study

Six to eight weeks female BALB/c nude mice were bought from the Institute of Laboratory Animal Science, Chinese Academy of Medical Sciences (CAMS) & Peking Union Medical College (PUMC). The mice were bred in cages with filter tops in a laminar flow hood in pathogen-free condition, with a 12 h light, 12 h dark cycle. All experimental procedures were approved by the Experimental Animal Care and Ethics Committee of the Institute of Microcirculation, CAMS & PUMC. MDA-MB-468/Roquin1-GFP (5 × 10^6^/100 μL PBS) and MDA-MB-468/GFP cells (5 × 10^6^/100 μL PBS) were injected subcutaneously into the back of nude mice, respectively. Then, tumor sizes were measured and recorded to draw the tumor growth curve. For tumor treatment with adenovirus, MDA-MB-231cells (3 × 10^6^/100 μL PBS) were injected into nude mice according to above methods. When tumors reached approximately 5 mm in diameter, GFP/Roquin1-expressing adenovirus or GFP-expressing control adenovirus (packaged at GeneChem, Shanghai) were injected into the tumors (10^10^ pfu/tumor each time) five times in total. Tumor size was measured by the formula length × width × high (mm^3^) in 60 days. Whole lung of nude mice was collected at the end of experiment and immersed in 10% formaldehyde solution.

### Cell lines and plasmids

The human breast cancer cell line (MDA-MB-231, MDA-MB-468, MCF7 and T47D), human normal mammary epithelial cell lines (MCF-10A and MCF-12A), human lung cancer cell line A549 cell, and human liver cancer cell line (HepG2) cells were obtained from the American Type Culture Collection (ATCC) and cultured in DMEM or RPMI-1640 with 10% FBS plus 1% Peni/Stro, respectively. HEK293 and HEK293T cells were obtained from National Infrastructure of Cell Line Resource (Beijing, China). The human full-length Roquin1 coding sequence (NM_172071) was synthesized, sequenced and inserted into pEGFP-N1 vector at EcoR I and Age I sites. Roquin1 serial deletion plasmids were generated by inserting the PCR-amplified fragments into pEGFP-N1 vector at EcoR I and Age I sites. A set of luciferase reporters were constructed by inserting the full-length 3’UTRs of human *MCM2*, *Cyclin D1*, and *Cyclin E1* or part 3’UTR (1-1440 bp) of *CDK6* into the pGL3 control vector (Promega) between Xba I and Fse I sites, respectively. For stem-loop deletion reporters, point mutated and truncated *CCNE1*–3’UTR (1-256 bp) (∆stem-loop) and *MCM2*–3’UTR (1–360 bp) (∆stem-loop) were amplified, sequenced, and inserted into pGL3 control vector using Phusion Site-Directed Mutagenesis Kit (Thermo Scientific). For stem-loop insertion constructs, the stem-loop sequences of *CCNE1* 3’UTR (257–276) and *MCM2* 3’UTR (361–377) were inserted into pGL3-β-actin^3’UTR^ reporter at 555 base pair.

### Cell cycle analysis

5 × 10^5^ cells were harvested and washed twice with PBS, and then fixed in cold ethanol (70%). The cells were then stained with propidium iodide (20 μg/mL) and RNase A (0.2 mg/mL) for 30 min. The stained cells (at least 1 × 10^4^ cells) were analyzed by flow cytometry and the data were analyzed with FlowJo software. All cell cycle analysis was performed in triplicate and repeated at least three times.

### RNA-sequencing analysis

MCF7/Roquin1-GFP, MDA-MB-468/Roquin1-GFP, A549/Roquin1-GFP, HepG2/Roquin1-GFP, and their control cells (expressing GFP) were cultured for 36 h and total RNA was extracted using the TRIzol method. RNA sequencing (RNA-seq) was completed by Allwegene Technology Inc., Beijing. The cDNA library was then constructed using polymerase chain reaction (PCR) amplification. RNA-seq was performed with the PE150 sequencing strategy using an Illumina second-generation high-throughput sequencing platform. RNA-seq reads with inferior quality or adapters were filtered. Clean read data were processed using Tophat2 and Cufflinks software to complete the alignment of transcriptomes. Genes not expressed in any sample were excluded from further analysis. Differentially expressed genes and transcripts were then filtered for false discovery rate (FDR)-adjusted *P* values less than or equal to 0.05.

### Gene ontology and Kyoto Encyclopedia of Genes and Genomes (KEGG) pathway analysis

RNA-seq data were deposited (PRJNA637876). The common up- and -downregulated mRNAs by Roquin1 in tumor cells were classified using the Venn diagram. Gene Ontology (GO) (biological process) and KEGG pathway analyses of commonly downregulated genes were done using DAVID Bioinformatics Tools and Ingenuity Pathway Analysis.

### RNA immunoprecipitation

Roquin1/GFP fusion protein was expressed in MDA-MB-468/Roquin1 cells, and then whole cell lysates were pre-cleared with isotype IgG, followed by incubation with anti-GFP antibody at 4 °C for 4 h. The protein-RNA complexes were then pulled down by protein G agarose beads (sc-2002, SantaCruz) and total RNA extracted with TRIzol, followed by detection of cell cycle-promoting genes with RT-PCR.

### Luciferase reporter assays

Luciferase assay was performed as described previously [24]. pGL3 luciferase reporter constructs containing full-length or segment of 3’UTR of different genes were transfected into HEK293 cells along with Roquin1/GFP, aa 1–441 (contain RING, ROQ, zinc finger domains), aa 441–1133 (contain PRD), aa 174–326 (only contain ROQ domain), and GFP-control constructs, respectively. All transfections were conducted in triplicate and repeated at least three times. The luciferase activity was measured 36 h after transfection using a Dual-Luciferase Reporter Assay System (Promega).

### mRNA stability

Roquin1/GFP was expressed in MDA-MB-468 cells, and then actinomycin D (ActD, 5 μg/mL) and 5, 6-dichlorobenzimidazole riboside (DRB, 5 μg/mL) were added to block de novo RNA synthesis. Total RNA was collected at indicated time points, and the relative mRNA level was analyzed by qPCR. The half-life of mRNA was determined by comparing with the levels of mRNA before adding ActD and DRB. The half-life of different genes in Roquin1 knockdown cells and their corresponding scrambled control cells was also tested as described earlier.

### shRNA lentivirus

Two lentiviral shRNAs (NM_172071.1–3458s1c1; NM_172071.1-2032s1c1) targeting human Roquin1 mRNA and two lentiviral shRNAs targeting human MCM2 (NM_004526.2-2553s21c1) and Cyclin E1 (NM_001238.1-1149s1c1) were purchased from Sigma. A scramble control shRNA was used as a control. Lentiviral particles were packaged in HEK293T cells by co-transfecting shRNA-pLKO.1, pCMV-dR8.2, and pMD2.G constructs. After 48 h, virus supernatants were collected and centrifuged to discard cell debris, and then added to target cells with 1 μg/mL polybrene for overnight. After two rounds infection, the target cells were selected with puromycin (2.5 μg/mL) for 2 weeks, followed by further study.

### Hematoxylin and Eosin (H&E staining)

Mouse lung tissues were immersed in 10% formalin for at least 2 weeks, and then stained with H&E staining. Zeiss Imaging System is used for visualizing of H&E sections.

### Statistical analysis

Data in bar graphs represent mean ± SD of at least three biological repeats. Statistical analysis was performed using Student’s t-test by comparing treatment versus vehicle control or otherwise as indicated. *P*-value < 0.05 was considered to be statistically significant.

## Results

### Roquin1 expression is reduced in breast cancer patients and is associated with poor survival

**Fig. 1:**
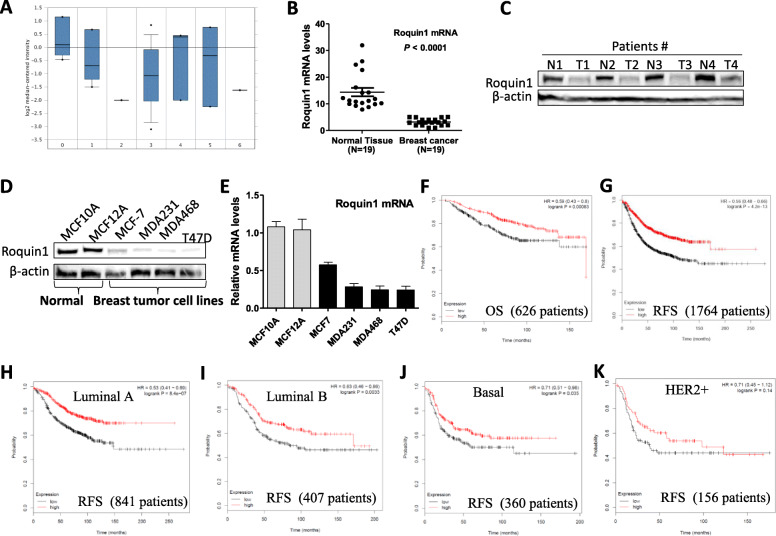
Roquin1 expression was reduced in breast cancer patients and was associated with poor survival. **a** Roquin1 mRNA expression among normal (0) (*n* = 4), breast carcinoma (1) (*n* = 4), ductal breast carcinoma in situ (2) (*n* = 1), invasive ductal breast carcinoma (3) (*n* = 18), invasive lobular breast carcinoma (4) (*n* = 3), invasive mixed breast carcinoma (5) (*n* = 3), and male breast carcinoma (6) (*n* = 1). **b** *Roquin1* expression was measured by qPCR in human breast tumor specimens (*n* = 19) compared with surrounding “normal” breast tissue (*n* = 19). **c** Roquin1 protein level was measured in human breast tumor tissues and normal mammary gland tissues. **d-e** Roquin1 protein (**d**) and mRNA (**e**) levels were measured by Western blot analysis and qPCR in human breast tumor cell lines and human normal mammary gland epithelial cell lines, respectively. **f-g** Kaplan–Meier overall survival (**f**) and relapse-free survival (**g**) curves of patients with breast tumors having low and high tumor Roquin1 transcripts. **h–k** Kaplan–Meier relapse-free survival curves of patients with luminal A (**h**), luminal B (**i**), basal (**j**), and Her2+ (**k**) breast tumor having low and high tumor Roquin1 transcripts

Roquin1 expression was first analyzed in a breast tumor database (www.oncomine.org). Low levels of Roquin1 were found in various breast cancers, including breast carcinoma, invasive ductal breast carcinoma, and invasive mixed breast carcinoma, although a moderately high level was found in invasive lobular breast cancer tissues (Fig. 1a; Additional file 1: Figure S1A). Experimentally, Roquin1 mRNA expression was significantly reduced in breast tumors compared with normal tissues (Fig. 1b). Roquin1 protein expression was also lower in four randomly selected pairs of breast cancer tissues than in their surrounding normal tissues (Fig. 1c). Moreover, Roquin1 expression was significantly repressed at both the protein (Fig. 1d) and mRNA (Fig. 1e) levels in several human breast cancer cell lines compared with normal mammary gland epithelial cells. Notably, by surveying Roquin1 expression across a gene array dataset [23], we found that low Roquin1 expression in human breast tumor samples was strongly associated with poor overall survival and relapse-free survival of patients (Fig. 1f and g). Furthermore, the levels of Roquin1 in breast tumors were associated with patient survival in the luminal A, luminal B, and basal-like subsets (Fig. 1h-j). Although no significant correlation was found between Roquin1 expression and patient survival in the HER2+ subsets, a similar trend in three other subsets was found (Fig. 1k). These results suggested that Roquin1 was important for the prognosis of patients with breast cancer. In addition, we found that Roquin1 was suppressed in other types of human cancers, including lung cancer, ovarian cancer, gastric cancer, and bladder carcinoma (Additional file 1: Figure S1B-1E). Roquin1 expression levels were also significantly correlated with the prognosis of patients with these cancers and liver cancer (Additional file 1: Figure S1F-1 J), indicating Roquin1 might be clinically predictive for multiple cancers.

### Roquin1 inhibits cell growth by inducing G1/S phase cell cycle arrest in tumor cells

**Fig. 2:**
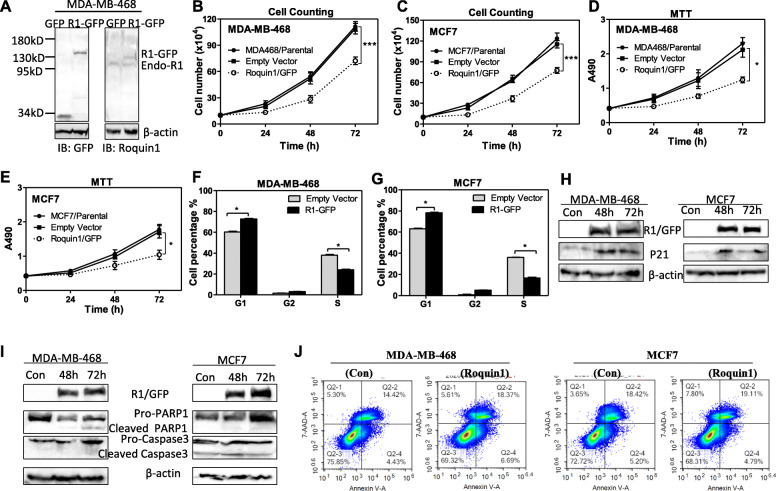
Roquin1 inhibits cell proliferation and induced G1/S phase cell cycle arrest in breast tumor cells. **a** Roquin1/GFP fusion protein was detected by immunoblotting with anti-GFP and anti-Roquin1 antibodies, respectively. **b-c** Cell counting were conducted to measure the growth of MDA-MB-468 (**b**) and MCF7 (**c**) cells after the overexpression of Roquin1/GFP (*n* = 3). ***P < 0.0001. **d-e** MTT assay was performed to measure the cell activity of MDA-MB-468 (**d**) and MCF7 (**e**) cells overexpressing Roquin1/GFP protein (*n* = 3). *P < 0.05. **f-g** Cell cycle was analyzed by FCM in MDA-MB-468 (**f**) and MCF7 (**g**) cells after Roquin1 overexpression. The proportions of cells in the G1, G2, and S phases are shown (*n* = 3). *P < 0.05. **h** p21 protein was detected by immunoblotting with an anti-p21 antibody at different time points after Roquin1 overexpression in MDA-MB-468 and MCF-7 cells. **i** Apoptosis indicators PARP-1 and caspase-3 were measured by immunoblotting at different time points after Roquin1 overexpression in MDA-MB-468 and MCF-7 cells. **j** Flow cytometric analyses of apoptotic cells in MDA-MB-468 and MCF7 cells with Roquin1 overexpression compared with their control cells

For analysis of Roquin1 function in breast cancer progression, the Roquin1/GFP fusion protein was expressed in MCF7 and MDA-MB-468 cells and identified by Western blotting (Fig. 2a). When Roquin1 was overexpressed, we found that the proliferation (Fig. 2b, c) and activity (Fig. 2d, e) of the tumor cells were substantially reduced. Similar results were also found in A549 and HepG2 with Roquin1 overexpression (Additional file 1: Figure S2A-2D). To determine whether Roquin1 inhibited cell proliferation by affecting tumor cell cycle progression, we evaluated the effect of Roquin1 overexpression on the cell cycle by flow cytometry (FCM). The G1 phase percentage of breast tumor cells was significantly increased in the Roquin1-overexpressing cancer cells compared with the controls. Moreover, a significant decrease in S phase percentage was detected after Roquin1 overexpression (Fig. 2f, g; Additional file 1: Figure S2E-2F). Similar results were also found in the A549 and HepG2 cells overexpressing Roquin1 (Additional file 1: Figure S2G-2 J). However, the percentage of cells in G2 phase cells did not change consistently among the tumor cells, which might be due to different cell types. These findings suggested that Roquin1 could induce G1/S cell cycle arrest in breast tumor cells. Indeed, the protein levels of p21, a typical cell cycle inhibitor, were induced by Roquin1 in tumor cells (Fig. 2h; Additional file 1: Figure S2K-2 L). To determine whether Roquin1 induced apoptosis in breast tumor cells, we detected cleaved caspase3 and PARP1, two key apoptotic indicators, by Western blotting. Roquin1 could not induce significant cleavage of pro-caspase3 and pro-PARP1 in breast tumor cells, although cleaved PARP1 was detected in MDA-MB-468 cells 72 h after Roquin1 overexpression (Fig. 2i). Our FACS data also showed that Roquin1 did not cause cell apoptosis in breast tumor cells (Fig. 2j). Collectively, these data clearly demonstrated that Roquin1 induces G1/S cell cycle arrest in breast tumor cells.

### Roquin1 selectively inhibits the mRNA expression of cell cycle–promoting genes by targeting 3’UTRs

**Fig. 3:**
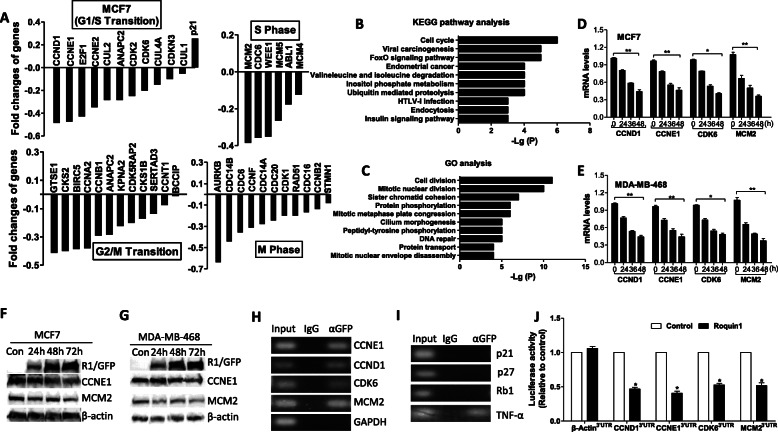
Roquin1 selectively inhibited the mRNA expression of cell cycle–promoting genes via targeting the 3′UTR. **a** RNA-seq analysis showed changes in the expression levels of cell cycle–related genes by Roquin1 in MCF7 cells. The genes promoting G1/S, S phase, G2/M, and M phase transition were downregulated; and the expression of cell cycle progression inhibitor p21 was upregulated. **b** Top 10 KEGG pathways enriched for common downregulated genes of Roquin1. **c** Top 10 GO terms (biological processes) for the analysis for common downregulated genes. **d-e** The mRNA levels of indicated cell cycle–promoting genes were measured by qPCR in MCF7 (**d**) and MDA-MB-468 (**e**) cells at indicated time points. *P < 0.05; **P < 0.01 between two groups. **f-g** CCNE1 and MCM2 were measured by immunoblotting with anti-CCNE1 and anti-MCM2 antibodies at different time points after Roquin1 overexpression in MCF7 (**f**) and MDA-MB-468 (**g**) cells. **h-i** Roquin1/GFP fusion protein were expressed in MDA-MB-468 cells, after lysate extraction, immunoprecipitation with anti-GFP antibody or IgG, and RNA extraction. Cell cycle–promoting gene (h) and –inhibiting gene (**i**) transcripts were detected by RT-PCR. **j** Measurement of luciferase activity of reporters containing the 3′UTRs of *CCND1, CCNE1, CKD6 (part),* and *MCM2,* respectively. β-Actin 3′UTR was used as a negative control. The results shown represent the mean ± standard deviation of four independent experiments. *P < 0.05

Next, we identified the genes affected by Roquin1 using RNA-seq in Roquin1-overexpressing MCF7 and MDA-MB-468 cells. Venn diagrams showed that 6556 genes were commonly downregulated and 7067 genes were commonly upregulated in two breast tumor cell lines (Additional file 1: Figure S3A). We further focused on the expression of cell cycle–related genes. Interestingly, the genes that promote cell cycle progression, including G1/S transition, G2/M transition, S phase transition, and M phase transition, were suppressed, whereas the genes inhibiting the cell cycle (*p21* and *Rb1*) were enhanced by Roquin1 in MCF7 (Fig. 3a) and MDA-MB-468 cells (Additional file 1: Figure S3B). Similar trends were also found in A549 and HepG2 cells (Additional file 1: Figure S3C-3D), indicating that Roquin1 could regulate the expression of cell cycle-related genes in tumor cells. Detailed RNA-seq data are summarized in Additional file 3: Table S1. Moreover, the ‘cell cycle’ pathway was the first of the top 10 signaling pathways significantly enriched in the KEGG pathway analysis of downregulated genes (Fig. 3b). The cell cycle–related terms ‘cell division’ and ‘mitotic nuclear division’ were enriched in the Gene Ontology (GO) analysis of downregulated genes (Fig. 3c). These computational analyses further supported our experimental findings. To validate the RNA-seq data, we measured four downregulated cell cycle–promoting genes (*CCND1*, *CCNE1*, *CDK6*, and *MCM2*) and three upregulated cell cycle–inhibiting genes (*p21*, *p27*, and *Rb1*) by real-time PCR. The mRNA expression of the four cell cycle–promoting genes was reduced in a time-dependent manner by Roquin1 in tumor cells (Fig. 3d, e). Additionally, the protein levels of CCNE1 and MCM2 were downregulated by Roquin1 over time (Fig. 3f, g). However, the upregulated cell cycle–inhibiting genes did not exhibit time-dependent changes (Additional file 1: Figure S3E). Notably, no time-dependent changes in the protein levels of p21 were observed in breast tumor cells (Fig. 2h, i). These results confirmed our RNA-seq data. Consistent with the overexpression results, the cell cycle–promoting genes were upregulated in the Roquin1^San/San^ MEF cells (Additional file 1: Figure S3F) [25], which further strengthened our findings. Taken together, these results indicate that Roquin1 regulates the cell cycle pathway by inhibiting the mRNA expression of cell cycle-promoting genes.

We next examined whether Roquin1 binds to the mRNAs of these cell cycle–promoting genes as an RBP. An RNA pull-down assay was performed with an anti-GFP antibody in the Roquin1/GFP-expressing MDA-MB-468 cells, followed by detection of the bound mRNAs by RT-PCR. The four cell cycle–promoting genes were amplified by PCR, whereas GAPDH and the cell cycle–inhibiting mRNAs were not amplified (Fig. 3h, i). *TNFα* was used as a positive control. These results indicated that Roquin1 selectively bound to the cell cycle–promoting genes but not the cell cycle–inhibiting genes. To determine whether mRNA binding was mediated through the 3’UTR, we cloned the 3’UTRs of *CCNE1*, *CCND1*, *CDK6* (part), and *MCM2* downstream of the luciferase gene as previously described [24] and then cotransfected these reporters with the Roquin1 expression vector and its empty vector into HEK293 cells, followed by the measurement of luciferase activity. As shown in Fig. 3j, Roquin1 significantly inhibited the luciferase activities of all four 3’UTR reporters compared with those of the cells transfected with control vector. The β-actin 3’UTR was used as a negative control. Collectively, these results suggested that Roquin1 specifically suppressed the mRNA expression of cell cycle–promoting genes by targeting their 3’UTRs.

### Roquin1 destabilizes the mRNAs of cell cycle–promoting genes via the ROQ domain

**Fig. 4:**
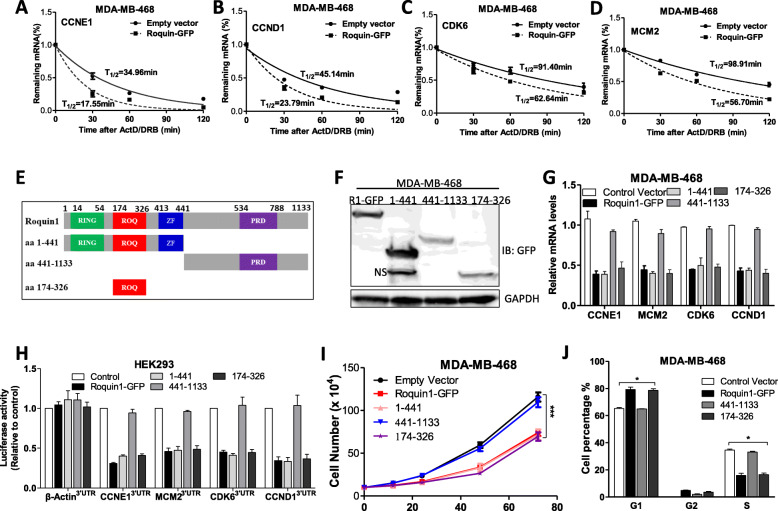
Roquin1 destabilized the mRNAs of cell cycle–promoting genes via the ROQ domain. **a–d** MDA-MB-468 cells overexpressing Roquin1/GFP protein were added with ActD and DRB for different time points. The levels of remaining cell cycle–promoting gene transcripts, including *CCND1* (**a**), *CCNE1* (**b**), *CDK6* (**c**), and *MCM2* (**d**), were measured by qPCR. **e** Schematic representation of the ROQ, RING, zinc finger (ZF), and PRD domains in Roquin1, and their truncations: aa 1–441, aa 441–1133, and aa 174–326. **f** Expression of Roquin1/GFP and its truncated mutations were confirmed by immunoblotting with an anti-GFP antibody. **g** MDA-MB-468 cells were transiently transfected with Roquin1 and its mutants. After 36 h, total RNA was extracted to measure the mRNA expression of indicated genes by qPCR. **h** HEK293 cells were co-transfected with indicated reporters and Roquin1 as well as its truncations. After 36 h, luciferase activity was measured in cell lysates and compared with that cells transfected with empty vector. **i** Cell counting was conducted in MDA-MB-468 cells after overexpressing Roquin1 and its mutants (*n* = 3). ***P < 0.0001. **j** Cell cycle was examined by FCM in MDA-MB-468 cells after overexpressing Roquin1 and its mutants (*n* = 3). *P < 0.05

We speculated that Roquin1 might reduce cell cycle–promoting genes by destabilizing their mRNAs. For confirmation of this hypothesis, Roquin1/GFP was expressed in MDA-MB-468 cells and then de novo mRNA synthesis was blocked using ActD (5 μg/mL) and DRB (5 μg/mL), followed by the measurement of the remaining mRNAs at different time points. The half-lives of indicated cell cycle–promoting mRNAs were shortened approximately 2-fold in Roquin1-overexpressing cells compared with the cells expressing the empty vector (Fig. 4a-d), while the half-lives of cell cycle–inhibiting mRNAs (including *p21*, *Rb1*, and *p27*) were barely affected by Roquin1 (Additional file 1: Figure S4A-4C), demonstrating that Roquin1 indeed inhibits cell cycle–promoting genes through mRNA stability.

The Roquin1 protein contains a RING finger, a ROQ domain, a zinc finger (ZF), and a proline-rich domain (PRD), and the ROQ domain is involved in the destabilization of mRNAs [10]. To determine whether the ROQ domain is also responsible for cell cycle–promoting mRNA decay, we generated a series of truncated Roquin1 mutants, including aa (amino acid) 1–441 containing the RING, ROQ, and ZF domains, aa 441–1133 containing the PRD domain, and aa 174–326 containing the ROQ domain (Fig. 4e), and identified by Western blot analysis (Fig. 4f). Then, the mutants were cotransfected with wild-type (WT) Roquin1 as well as different 3’UTR reporters (Additional file 1: Figure S4D) into HEK293 cells. As shown in Fig. 4g and h, the WT and the mutants aa 1–441 and aa174–326, but not the mutant aa 441–1133, suppressed the mRNA expression of four cell cycle–promoting genes and the luciferase activities of their 3’UTR reporters, which was also consistent with a previous report [26]. In addition, aa 174–326 significantly inhibited the proliferation (Fig. 4i) and cell cycle progression (Fig. 4j; Additional file 1: Figure S4E) of MDA-MB-468 cells, indicating that the ROQ domain in Roquin1 is essential for the induction of breast tumor cell cycle arrest.

### Roquin1 knockdown stabilizes cell cycle–promoting gene transcripts and promotes tumor cell cycle progression

**Fig. 5:**
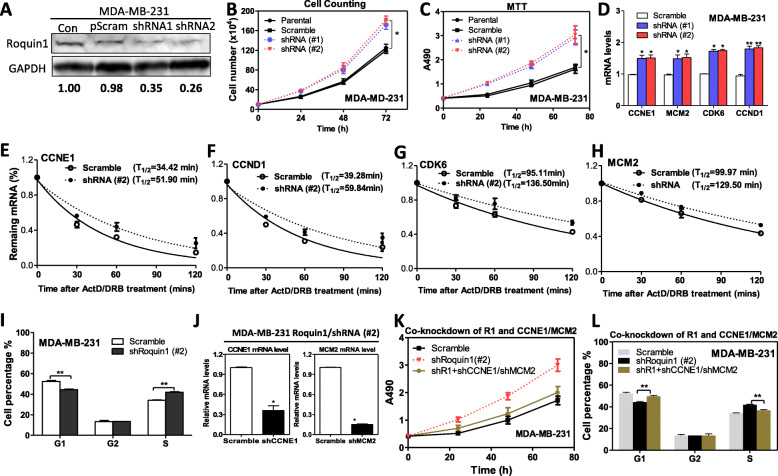
Roquin1 knockdown stabilized cell cycle–promoting gene transcripts and promoted G1/S cell cycle progression. **a** Roquin1 knockdown was validated by western blot analysis. **b-c** Cell proliferation was measured by cell counting (**b**) and MTT assay (**c**) in MDA-MB-231 cells after knocking down Roquin1 at the indicated time points (*n* = 3). *P < 0.05. **d** Cell cycle–promoting gene mRNAs were measured in the Roquin1 knocked down MDA-MB-231 cells by qPCR. **P < 0.01 between two groups. **e–h** The half-lives of cell cycle–promoting genes were measured in Roquin1 knockdown MDA-MB-231 cells. **i** Cell cycle analysis was performed in MDA-MB-231 cells after the knockdown of Roquin1. The proportions of cells in the G1, G2, and S phases are shown (*n* = 3). **P < 0.01. **j** MDA-MB-231/Roquin1-knockdown cells were infected with scramble/lentivirus or shRNA lentivirus targeting CCNE1 and MCM2, respectively. Total RNA was extracted to measure the levels of mRNAs of CCNE1 and MCM2. *P < 0.05. **k** MTT assay was conducted to measure the cell growth of MDA-MB-231 cells after the co-knockdown of Roquin1 and CCNE1/MCM2. **l** Cell cycle analysis was performed in MDA-MB-231 cells after the co-knockdown of Roquin1 and CCNE1/MCM2. **P < 0.01

To further confirm the inductive effects of Roquin1 on tumor cell cycle arrest, we suppressed Roquin1 expression with two shRNAs in MDA-MB-231 cells, another triple-negative breast cancer cell line. Roquin1 was reduced by approximately 65 and 74% by #1shRNA and #2shRNA, respectively (Fig. 5a). Although Roquin1 is expressed at low levels in breast tumors, the knockdown of Roquin1 strongly promoted the proliferation and activities of breast tumor cells (Fig. 5b, c) and increased the mRNA expression of cell cycle–promoting genes (Fig. 5d). However, depletion of Roquin1 had no effect on the mRNA levels of p21, Rb1, and p27 (Additional file 1: Figure S5A), again suggesting that Roquin1 directly suppressed the mRNA expression of cell cycle–promoting genes. Next, we examined the effect of Roquin1 knockdown on the half-life of cell cycle–promoting genes. As expected, reduced Roquin1 significantly prolonged the half-lives of the indicated cell cycle–promoting mRNAs (Fig. 5e-h). Furthermore, we found a reduced percentage of G1 phase cells and an increased percentage of S phase MDA-MB-231 cells after Roquin1 knockdown (Fig. 5i; Additional file 1: Figure S5B). To confirm whether the cell cycle–promoting genes were involved in the Roquin1-induced cell cycle arrest, we knocked down CCNE1 and MCM2 by shRNA lentivirus in the Roquin1 knockdown MDA-MB-231 cells. Figure 5j shows that these shRNAs effectively knocked down *CCNE1* and *MCM2* expression. Upon co-knockdown of Roquin1 and CCNE1/MCM2, cell proliferation was closed to that of the scramble control compared to Roquin1 knockdown alone (Fig. 5k). Additionally, the percentage of G1 phase cells was significantly increased compared with that of the group with Roquin1 knockdown alone, and the percentage of S phase cells significantly decreased (Fig. 5l). Collectively, these results confirmed that Roquin1 repression indeed promotes breast tumor cell cycle progression by stabilizing cell cycle-promoting genes.

### Roquin1 binds to the stem–loop structure of cell cycle-promoting genes for degradation

**Fig. 6:**
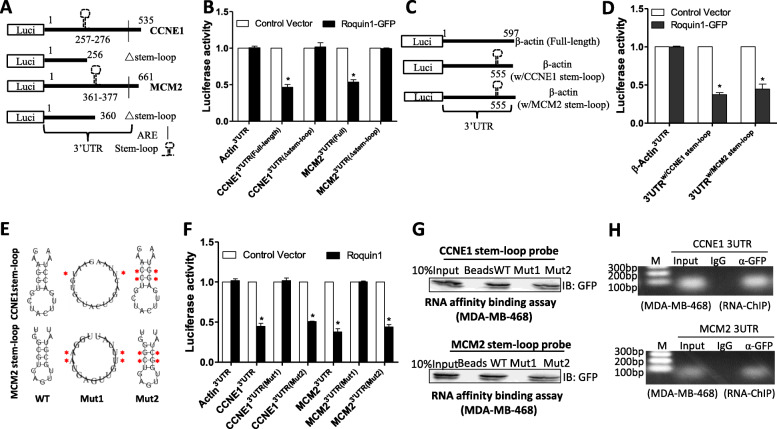
Roquin1 bound to the stem–loop structure in the 3′UTR of cell cycle–promoting genes. **a** Schematic representation of the luciferase reporter constructs of *CCNE1* and *MCM2* containing truncated 3′UTRs without the stem–loop structure (Δ stem–loop). **b** Measurement of luciferase activity of reporters containing full-length 3′UTRs or truncated 3′UTRs (Δ stem–loop) of *CCNE1* and *MCM2,* respectively. The results shown represent the mean ± standard deviation of four independent experiments. *P < 0.05. **c** Schematic representation of the luciferase reporter constructs of human β-actin 3′UTR containing the stem–loop structure of *CCNE1* (w/CCNE1 stem–loop) or *MCM2* (w/MCM2 stem–loop). **d** Measurement of luciferase activity of reporters containing wild-type β-actin 3′UTRs or β-actin 3′UTRs with stem-loop sequences of *CCNE1* and *MCM2*. *P < 0.05. **e** The predicted stem–loop structures of *CCNE1* (top) and *MCM2* (bottom) in their 3′UTRs and mutation strategy (asterisks indicate base substitution). Mutant1 was unable to form a stem–loop structure (middle), and Mutant2 still formed a stem–loop structure (left). **f** Luciferase assays were conducted using reporters from (E) along with Roquin1 or control vector, and then measurement of the luciferase activity. *P < 0.05. **g** Roquin1/GFP fusion protein was expressed in MDA-MB-468 cells for 24 h, and then cell lysates were collected. Biotinylated *CCNE1* and *MCM2* wild-type or mutant probes (mut1 and mut2) were used for the pull-down assay. **h** RIP-ChIP assay was conducted with genome fragments from MDA-MB-468 cells after Roquin1/GFP fusion protein expression for 24 h

Roquin1 is known to degrade target mRNAs by binding to the stem–loop structure [16]. The 3’UTR sequences of four cell cycle–promoting genes were analyzed, and a conserved sequence was identified across species, which could form a similar stem–loop structure (Additional file 1: Figure S6A-6D) using RNAfold WebServer [27]. To investigate the role of the stem–loop structure in Roquin1-mediated degradation of cell cycle–promoting mRNAs, we generated deletion constructs by deleting the sequences containing the stem–loop in the 3’UTRs of *CCNE1* and *MCM2* (Fig. 6a). Then, full-length and deletion reporters with Roquin1 were cotransfected into HEK293 cells, followed by measurement of luciferase activity. Roquin1 significantly inhibited the luciferase activity of the full-length *CCNE1* and *MCM2* 3’UTRs but not the deletion mutant reporters (Fig. 6b). In addition, Roquin1 reduced the activities of the reporters containing human β-actin 3’UTR with *CCNE1* or *MCM2* stem–loop structures compared with that in the control group (Fig. 6c, d). These findings indicated that the stem-loop structure was pivotal for Roquin1-mediated cell cycle–promoting mRNAs decay.

To determine the necessity of the stem–loop secondary conformation for mRNA degradation, we generated two 3’UTR mutant reporters of CCNE1 and MCM2; the stem–loop structure of mutant1 was deleted by replacing two or four nucleotides, and mutant2 retained the stem–loop structure after replacement of four nucleotides (Fig. 6e). Deletion of the stem–loop structure in the 3’UTRs of *CCNE1* and *MCM2* (mutant1) allowed them to be completely resistant to Roquin1 inhibition, while the mutant2 that maintained the stem–loop structure remained sensitive to Roquin1 suppression (Fig. 6f), indicating that the stem–loop structure in 3’UTRs was critical for cell cycle–promoting mRNAs decay. To further determine whether Roquin1 physically bound to the stem–loop in the 3’UTRs of *CCNE1* and *MCM2*, we performed an RNA affinity binding assay with biotin-labeled RNA probes. Wild-type RNA probes and mutant probes with the stem–loop structure either disrupted (mutant1) or retained (mutant2) were incubated with lysates of MDA-MB-468 cells expressing the Roquin1/GFP fusion protein. Then, streptavidin-coated magnetic beads were used for the pulldown assay, followed by Western blot detection with an anti-GFP antibody. The Roquin1/GFP fusion protein was pulled down by wild-type and mutant2 probes but not by the stem–loop structure-deficient mutant1 probe (Fig. 6g), indicating that Roquin1 indeed interacted with the stem–loop structure of *CCNE1* and *MCM2* in vitro. Furthermore, a modified RNA immunoprecipitation-chromatin immunoprecipitation (RIP-ChIP) assay was performed to verify that Roquin1 could bind the stem–loop structure in vivo. The Roquin1/GFP fusion protein was expressed in MDA-MB-468 cells, and the protein-RNA complex was pulled down by GFP antibody-coated beads after the bound mRNAs were sonicated, followed by amplification of the stem–loop sequences by RT-PCR. As expected, the stem–loop sequences in the 3’UTRs of *CCNE1* and *MCM2* could be amplified in the GFP antibody pulldown group but not in the group using isotype IgG (Fig. 6h), indicating the binding of Roquin1 to the 3’UTRs of cell cycle–promoting mRNAs in breast tumor cells. Overall, these data demonstrated that Roquin1 recognized and bound to the stem–loop structure in the 3′UTRs of cell cycle–promoting genes for degradation.

### Roquin1 suppresses breast tumor growth and metastasis

**Fig. 7:**
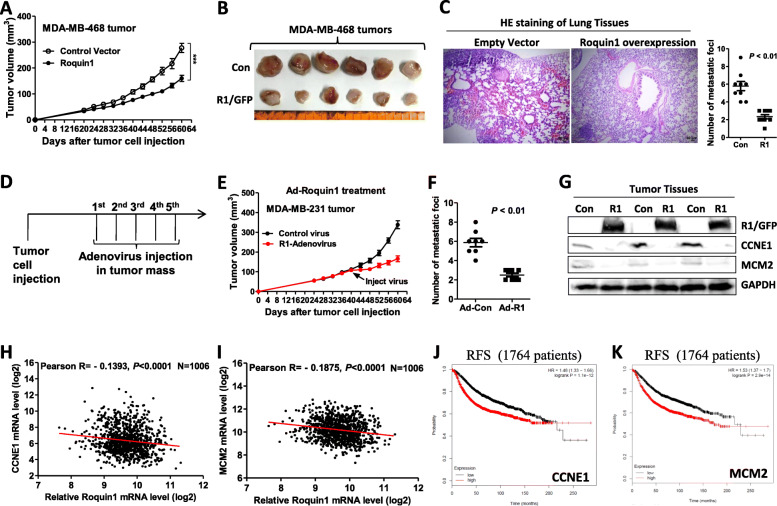
Roquin1 suppressed breast tumor growth and metastasis. **a** Tumor growth curves in nude mice received 5 × 10^6^ MDA-MB-468/Roquin-GFP and MDA-MB-468/GFP cells (*n* = 6/group). ***P < 0.0001 between two groups. **b** MDA-MB-468/GFP and MDA-MB-468/Roquin1/GFP tumors were collected and compared at the end of the experiment. **c** H&E staining of lung tissue sections from nude mice bearing MDA-MB-468/GFP or MDA-MB-468/Roquin1–GFP tumors. Scale bar, 50 μm. **d** Experimental flow chart for tumor treatment with Roquin1-expressing adenovirus in vivo. MDA-MB-231 cells (3 × 10^6^ cells/100 μL of PBS) were injected into nude mice to establish a tumor mass. Tumors were treated with adenovirus (10^9^ PFU adenovirus in 100 μL of PBS) every other day when tumors grew to a certain size (~ 5 mm in diameter). **e** Tumor growth curves in nude mice after treatment with adenovirus. Black arrows indicate the time point of adenovirus injection. **f** Quantification of the number of metastatic foci of each mouse treated with control or Roquin1-expressing adenovirus. **g** Total protein was extracted from tumor tissues to detect the expression of Roquin1/GFP, CCNE1, and MCM2 by immunoblotting with anti-GFP, anti-CCNE1, and anti-MCM2 antibodies, respectively. β-actin was used as a loading control. **h-i** Pearson’s Correlation analysis between *Roquin1* and *CCNE1* (**h**) and *MCM2* (**i**) expression levels in log2 values in human breast cancer patients (*n* = 1006). **j-k** Kaplan–Meier relapse-free survival curves of patients with breast cancer having low and high levels of tumor *CCNE1* (**j**) and *MCM2* (**k**) transcripts

To determine the inhibitory effect of Roquin1 on breast cancer progression in vivo, we inoculated MDA-MB-468/Roquin1-GFP cells (expressing the Roquin1/GFP fusion protein) and MDA-MB-468/GFP cells into the mammary gland fat pads of female nude mice. The growth and sizes of the tumors expressing the Roquin1/GFP fusion protein were significantly reduced compared with those of the control tumors (Fig. 7a, b). Roquin1/GFP fusion protein expression in tumors was confirmed by Western blot analysis (Additional file 1: Figure S7A). Moreover, a significant decrease in the number of metastatic foci (Fig. 7c) and metastatic white nodules (Additional file 1: Figure S7B) was observed in the lung tissues from Roquin1/GFP tumor-bearing mice. To avoid the impacts of the manual manipulation of gene expression and simulate the clinical treatment of breast cancer, we prepared adenoviruses expressing the Roquin1/GFP fusion gene and its control virus (expresses GFP) to treat the established MDA-MB-231 breast tumors in nude mice. When tumor mass reached approximately 5 mm in diameter, 10^10^ pfu of Roquin1/GFP adenovirus in 100 μL of PBS and the control adenovirus were injected every other day for five injections in total (Fig. 7d). Two days after injection, the tumors began to shrink and grew slowly, while the tumors treated with control adenovirus continued growing (Fig. 7e). At the end of the experiment, the sizes of tumors treated with the Roquin1/GFP adenovirus were significantly smaller than those in the control group (Additional file 1: Figure S7C). Tumor metastasis was also significantly suppressed by Roquin1 adenovirus treatment (Fig. 7f; Additional file 1: Figure S7D). Consistent with the in vitro results, the protein levels of CCNE1 and MCM2 were also reduced in the Roquin1 adenovirus-treated tumors (Fig. 7g), further confirming that Roquin1 suppressed the expression of cell cycle–promoting genes in vivo. Interestingly, the expression of *CCNE1* and *MCM2* was also significantly inhibited as *Roquin1* increased in 1006 human breast cancer samples (Fig. 7h, i) (Additional file 4: Table S2) (Oncolnc.org/). Notably, higher levels of *CCNE1* and *MCM2* negatively correlated with poor survival of patients with breast cancer (Fig. 7j, k). Conclusively, these findings strongly suggest that Roquin1 is a promising breast tumor suppressor and that the Roquin1-cell cycle-promoting gene axis might be considered a new therapeutic target for breast tumor treatment in the future.

## Discussion

Currently, the role of Roquin1 in tumor progression has been poorly studied. Analysis of the The Cancer Genome Atlas (TCGA) breast cancer databases showed that Roquin1 was expressed at low levels in human breast cancers. This low-level expression pattern was further confirmed in human breast cancer tissues and cell lines. In addition, low Roquin1 expression in tumors from various subtypes of breast cancer patients was strongly associated with poor survival, indicating the clinical significance of Roquin1. Despite no significant association in HER2+ breast cancer, the trend was similar to that of other subtypes. Besides, the levels of Roquin1 in several types of human cancers were associated with patient survival, indicating that Roquin1 might be important for the prognosis of multiple types of human cancers.

We initially focused on the changes of cytokines secreted by tumor cells following Roquin1 overexpression. Unexpectedly, we found that Roquin1 significantly suppressed cell proliferation and induced cell cycle arrest in vitro. To our knowledge, the relationship between Roquin1 and cell cycle progression has not been reported to date, especially in breast tumor progression. Using FCM, we found that Roquin1 could significantly inhibit the G1/S transition in breast cancer cells as well as lung and liver cancer cells. However, we did not observe obviously cell apoptosis after Roquin1 overexpression, which was different from the apoptotic induction role of another RBP, monocyte chemotactic protein-induced protein 1 (MCPIP1), in breast cancer progression [24]. Cell cycle arrest is triggered when the balance between cell cycle–promoting and cell cycle–inhibiting genes is disrupted. Our results clearly demonstrated that most of the genes that promoted cell cycle progression were downregulated by Roquin1, while those that inhibited cell cycle progression were upregulated. Indeed, four cell cycle-promoting genes, but not the cell cycle–inhibiting genes, were confirmed to be suppressed in a time-dependent manner. Selective targeting of Roquin1 to cell cycle–promoting mRNAs but not to cycle-inhibiting mRNAs might trigger an imbalance between cell cycle–promoting and inhibiting genes in tumor cells. Roquin1 also inhibited cell cycle progression in human liver cancer cells and lung cancer cells, indicating that Roquin1 might elicit an antitumor response in a wide range of human cancers.

Among the Roquin1 target genes, *CCNE1*, *CCND1*, and *CDK6* are involved mainly in the G1/S transition [28–30], while *MCM2* is important for DNA replication and the S phase transition of the cell cycle [31]. The broad targets of Roquin1 in the cell cycle pathway suggest that Roquin1 induces cell cycle arrest by targeting multiple molecules in breast tumor cells. We showed that Roquin1 suppresses the expression of these genes at the post-transcriptional level by enhancing mRNA degradation. The 3′UTR is important for the post-transcriptional regulation of genes. Many RBPs, including human antigen R (HuR) [32], tristetraprolin (TTP) [33], and MCPIP1, have been reported to regulate mRNA stability by the 3’UTR. Indeed, Roquin1 inhibits luciferase activity through the 3’UTRs of cell cycle–promoting genes. It has been reported that the ROQ domain is essential for Roquin1-mediated mRNA degradation and immune regulatory effects [34]. We demonstrated that the ROQ domain is also required for the expression of cell cycle–promoting genes and cell cycle arrest induction by mutating the domains of Roquin1.

Many elements responsible for mRNA degradation are primarily localized in the 3’UTR, such as ARE, GU-rich element (GRE), and stem–loop structure [35–37]. Roquin is known to recruit other deadenylases to the 3’UTR for RNA decay by recognizing and binding the stem–loop structure [38]. Our results demonstrated that the stem–loop structure but not the ARE in the 3’UTRs is required for Roquin1-mediated cell cycle–promoting mRNAs decay. Roquin1 has been shown to bind a constitutive decay element (CDE) in the 3’UTR of TNFα mRNA, and this CDE can fold into a stem–loop structure [39]. Indeed, we found that a conserved consensus sequence was shared among different specifies in four cell cycle–promoting genes, and these sequences could fold into a stem–loop structure. Strikingly, no common stem–loop sequences were identified among the four cell cycle–promoting genes, which supports our hypothesis that Roquin1 mainly recognizes the secondary structure instead of the linear sequence in the 3′UTR, which was also consistent with previous findings [15].

Finally, we proposed a model to elucidate the potential role of Roquin1 in the suppression of cell cycle progression (Additional file 1: Figure S7E). Roquin1 selectively targets the mRNAs of cell cycle–promoting gene for degradation via its ROQ domain by binding to the stem–loop structures in the 3’UTRs. As an RBP located in the cytoplasm, Roquin1 can regulate cell cycle progression by balancing the expression of cell cycle–related genes in tumor cells. Moreover, Roquin1 expression was significantly negatively correlated with the expression of cell cycle–promoting genes in human breast tumors, further demonstrating the biological relevance of Roquin1 and the suppression of breast cancer. Our findings provide a novel regulator for the cell cycle signaling pathway and identify new target genes of Roquin1.

## Conclusions

In summary, this study demonstrates that Roquin1 implicates in regulating the growth and metastasis of breast cancer by inhibiting cell cycle progression and proliferation. Roquin1 disrupts the balance of the cell cycle signaling pathway by directly binding and destabilizing cell cycle-promoting genes via the ROQ domain, which ultimately induced G1/S cell cycle arrest in cancer cells. Notably, low Roquin1 expression in breast tumors is strongly associated with poor survival of patients with breast cancer. Therefore, Roquin1 might be a new cell cycle suppressor in breast cancer, which could be a promising molecular target for tumor treatment.

## Supplementary Information

**Additional file 1:** **Figure S1.** Roquin1 expression is reduced in several human cancers, and positively associated with patient survival. a Comparison of Roquin1 mRNA expression between normal (0) (*n* = 7) and ductal breast carcinoma (1) (*n* = 40). b Comparison of Roquin1 mRNA expression among normal (0) (*n* = 65), large cell lung carcinoma (1) (*n* = 19), lung adenocarcinoma (2) (*n* = 45), and squamous cell lung carcinoma (3) (*n* = 27). c Comparison of Roquin1 mRNA expression among normal (0) (*n* = 5), ovarian clear cell large cell adenocarcinoma (1) (*n* = 7), ovarian endometrioid adenocarcinoma (2) (*n* = 9), ovarian mucinous adenocarcinoma (3) (*n* = 9), and ovarian serous adenocarcinoma (4) (*n* = 20). d Comparison of Roquin1 mRNA expression in normal (0) (*n* = 31), diffuse gastric adenocarcinoma (1) (*n* = 6), and gastric adenocarcinoma (2) (*n* = 2). e Comparison of Roquin1 mRNA expression in normal (0) (*n* = 3), bladder cancer (1) (*n* = 3), bladder squamous cell carcinoma (2) (*n* = 1), bladder urothelial carcinoma (3) (*n* = 3), infiltrating bladder urothelial carcinoma (4) (*n* = 34), bladder papillary urothelial carcinoma (5) (*n* = 1), and superficial bladder cancer (6) (*n* = 17). f-j Kaplan-Meier overall survival curve of patients with lung cancer (f), ovarian cancer (g), gastric cancer (h), bladder carcinoma (i), and liver cancer (j) having low and high tumor Roquin1 transcripts. **Figure S2.** Roquin1 inhibits cell proliferation and induces G1/S phase cell cycle arrest in tumor cells. a-b Cell counting was carried out every 24 h in A549 (a) and HepG2 (b) cells with Roquin1/GFP overexpression. ***P < 0.001. c-d MTT assay was performed in A549 and HepG2 cells to measure cell proliferation after Roquin1 overexpression. *P < 0.05. e-h Representative cell cycle histograms showing cell cycle analyses of MDA-MB-468 (e), MCF7 (f), A549 (g), and HepG2 (h) cells after Roquin1 overexpression. i-j Cell cycle analysis was carried out in A549 (i) and HepG2 (j) cells after Roquin1 overexpression, and the percentages of different cell phases were quantified. **P < 0.01. k-l The protein level of cell cycle inhibitor p21 was measured by immunoblotting with an anti-p21 antibody at different time points after Roquin1 overexpression in A549 (k) and HepG2 (l) cells. β-actin was used as a loading control. **Figure S3.** Roquin1 selectively inhibits the mRNA expression of cell cycle-promoting genes. a Venn diagrams showing the common down-regulated (upper) and up-regulated genes (bottom) by Roquin1 in breast tumor cells. b-d The expression levels of cell cycle–related genes affected by Roquin1 were analyzed by RNA-seq in MDA-MB-468 (b), A549 (c), and HepG2 (d) cells. The expression of genes promoting G1/S, S phase, G2/M, and M phase transition was downregulated; and the expression of cell cycle–inhibiting genes were upregulated. e The mRNA expression levels of indicated cell cycle–inhibiting genes were measured by qPCR in MDA-MB-468 and MCF7 cells at different time points, including *p21*, *p27*, *p16*, and *Rb1*. f Cell cycle–related genes were regulated by Roquin1 in Roquin1^san/san^ MEF cells. The expression of genes promoting G1/S, S phase, G2/M, and M phase transition were upregulated. **Figure S4.** Roquin1 destabilizes the mRNAs of cell cycle-promoting genes via the ROQ domain. a–c The half-lives of cell cycle–inhibiting genes, including *p21* (a)*, p16* (b)*,* and *p27* (c) were measured by qPCR in Roquin1-expressing MDA-MB-468 cells. d Schematic representation of the luciferase reporter constructs containing 3’UTRs sequences of *CCNE1*, *CCND1*, *CDK6* (part), and *MCM2*. e Representative cell cycle histograms showing cell cycle analyses of MDA-MB-468 cells after overexpression of Roquin1/GFP, aa 441–1131, and aa 174–326 truncated mutations. **Figure S5.** Knocking down Roquin1 enhances breast tumor cell cycle progression. a The mRNA expression levels of indicated cell cycle–inhibiting genes were measured after Roquin1 knockdown by infecting lentivirus expressing shRNA/Scramble and shRNA/Roquin1 by qPCR in MDA-MB-231 cells*.* b Representative cell cycle histograms showing cell cycle analyses of MDA-MB-231 cells after knocking down Roquin1. **Figure S6.** Putative stem-loop structure in the 3’UTRs of cell cycle-promoting genes. a–d The 3’UTR sequences from different species for each cell cycle–promoting gene, including *CCNE1* (a), *MCM2* (b), *CDK6* (c), and *CCND1* (d), was aligned using DNAMAN software. The stem-loop sequences were predicted by RNAfold web server to fold a secondary stem–loop structure (right) and indicated by red box. **Figure S7.** Roquin1 suppresses breast tumor growth and metastasis. a Total protein was extracted from tumor tissues and used to detect Roquin1/GFP expression by immunoblotting with an anti-GFP antibody. b Whole lungs from nude mouse bearing MDA-MB-468/GFP or MDA-MB-468/Roquin1/GFP tumors was collected and compared. c MDA-MB-231 tumors treated with control adenovirus (Ad-GFP) or Roquin1-expressing adenovirus (Ad-R1/GFP). d H&E staining of lung sections of tumor-bearing mice treated with control adenovirus or Roquin1-expressing adenovirus. Scale bar, 50 μm. e A proposed work model of cell cycle-promoting genes regulation by Roquin1.

**Additional file 2.**

**Additional file 3:** **Supplemental Table 1.** RNA-seq analysis of human tumor cells overexpressing Roquin1.

**Additional file 4.**

**Additional file 5:** **Supplementary Table S3.** List of primer and RNA-EMSA probes sequences used in this study.

## Data Availability

All data generated or analyzed during this study are included either in this article or in the supplementary information files.

## Abbreviations

MTT: Thiazolyl Blue Tetrazolium Bromide
FCM: Flow cytometry
RIP-ChIP: RNA immunoprecipitation-chromatin immunoprecipitation
CCND1: Cyclin D1
CCNE1: Cyclin E1
CDK6: Cyclin dependent kinase 6
MCM2: Minichromosome maintenance 2
3’UTR: 3′ untranslated region
RBP: RNA-binding protein
RING: Really Interesting New Gene
ZF: Zinc finger
ROQ: ROQ domain containing RNA binding activity
ATCC: American Type Culture Collection
RPMI 1640: Roswell Park Memorial Institute 1640
shRNA: Short hairpin RNA
DMEM: Dulbecco’s Modified Eagle’s Medium
DRB: 5, 6-dichlorobenzimidazole riboside
ActD: Actinomycin D
ARE: AU-rich element
MEF: Mouse Embryonic Fibroblast
