# Supplementary material for: Roquin1 inhibits the proliferation of breast cancer cells by inducing G1/S cell cycle arrest via selectively destabilizing the mRNAs of cell cycle–promoting genes
Source: J Exp Clin Cancer Res. 2020 Nov 23;39:255. doi: 10.1186/s13046-020-01766-w (PMC7686734; doi:10.1186/s13046-020-01766-w)
Supplement: Supplementary file 1 — Additional file 1: Figure S1. Roquin1 expression is reduced in several human cancers, and positively associated with patient survival. a Comparison of Roquin1 mRNA expression between normal (0) (n = 7) and ductal breast carcinoma (1) (n = 40). b Comparison of Roquin1 mRNA expression among normal (0) (n = 65), large cell lung carcinoma (1) (n = 19), lung adenocarcinoma (2) (n = 45), and squamous cell lung carcinoma (3) (n = 27). c Comparison of Roquin1 mRNA expression among normal (0) (n = 5), ovarian clear cell large cell adenocarcinoma (1) (n = 7), ovarian endometrioid adenocarcinoma (2) (n = 9), ovarian mucinous adenocarcinoma (3) (n = 9), and ovarian serous adenocarcinoma (4) (n = 20). d Comparison of Roquin1 mRNA expression in normal (0) (n = 31), diffuse gastric adenocarcinoma (1) (n = 6), and gastric adenocarcinoma (2) (n = 2). e Comparison of Roquin1 mRNA expression in normal (0) (n = 3), bladder cancer (1) (n = 3), bladder squamous cell carcinoma (2) (n = 1), bladder urothelial carcinoma (3) (n = 3), infiltrating bladder urothelial carcinoma (4) (n = 34), bladder papillary urothelial carcinoma (5) (n = 1), and superficial bladder cancer (6) (n = 17). f-j Kaplan-Meier overall survival curve of patients with lung cancer (f), ovarian cancer (g), gastric cancer (h), bladder carcinoma (i), and liver cancer (j) having low and high tumor Roquin1 transcripts. Figure S2. Roquin1 inhibits cell proliferation and induces G1/S phase cell cycle arrest in tumor cells. a-b Cell counting was carried out every 24 h in A549 (a) and HepG2 (b) cells with Roquin1/GFP overexpression. ***P < 0.001. c-d MTT assay was performed in A549 and HepG2 cells to measure cell proliferation after Roquin1 overexpression. *P < 0.05. e-h Representative cell cycle histograms showing cell cycle analyses of MDA-MB-468 (e), MCF7 (f), A549 (g), and HepG2 (h) cells after Roquin1 overexpression. i-j Cell cycle analysis was carried out in A549 (i) and HepG2 (j) cells after Roquin1 overexpression, and the [file 13046_2020_1766_MOESM1_ESM.docx]

**Additional file 1:**


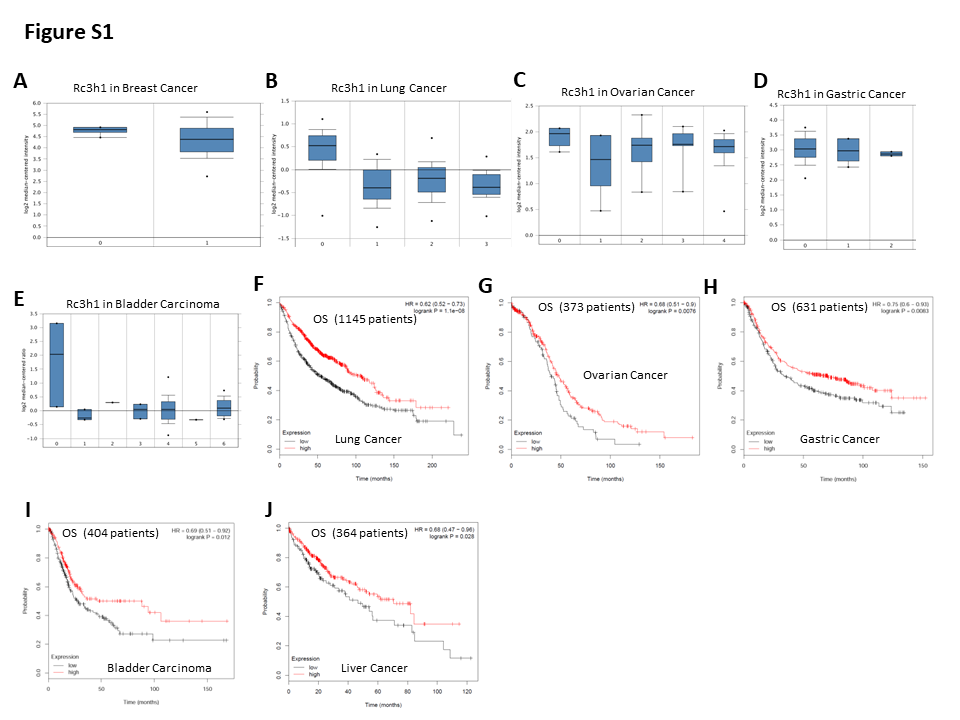


**Figure S1.** Roquin1 expression is reduced in several human cancers, and positively associated with patient survival. **a** Comparison of Roquin1 mRNA expression between normal (0) (*n* = 7) and ductal breast carcinoma (1) (*n* = 40). **b** Comparison of Roquin1 mRNA expression among normal (0) (n = 65), large cell lung carcinoma (1) (*n* = 19), lung adenocarcinoma (2) (*n* = 45), and squamous cell lung carcinoma (3) (*n* = 27). **c** Comparison of Roquin1 mRNA expression among normal (0) (*n* = 5), ovarian clear cell large cell adenocarcinoma (1) (*n* = 7), ovarian endometrioid adenocarcinoma (2) (*n* = 9), ovarian mucinous adenocarcinoma (3) (*n* = 9), and ovarian serous adenocarcinoma (4) (*n* = 20). **d** Comparison of Roquin1 mRNA expression in normal (0) (*n* = 31), diffuse gastric adenocarcinoma (1) (*n* = 6), and gastric adenocarcinoma (2) (*n* = 2). **e** Comparison of Roquin1 mRNA expression in normal (0) (*n* = 3), bladder cancer (1) (*n* = 3), bladder squamous cell carcinoma (2) (*n* = 1), bladder urothelial carcinoma (3) (*n* = 3), infiltrating bladder urothelial carcinoma (4) (*n* = 34), bladder papillary urothelial carcinoma (5) (*n* = 1), and superficial bladder cancer (6) (*n* = 17). **f-j** Kaplan-Meier overall survival curve of patients with lung cancer (**f**), ovarian cancer (**g**), gastric cancer (**h**), bladder carcinoma (**i**), and liver cancer (**j**) having low and high tumor Roquin1 transcripts.


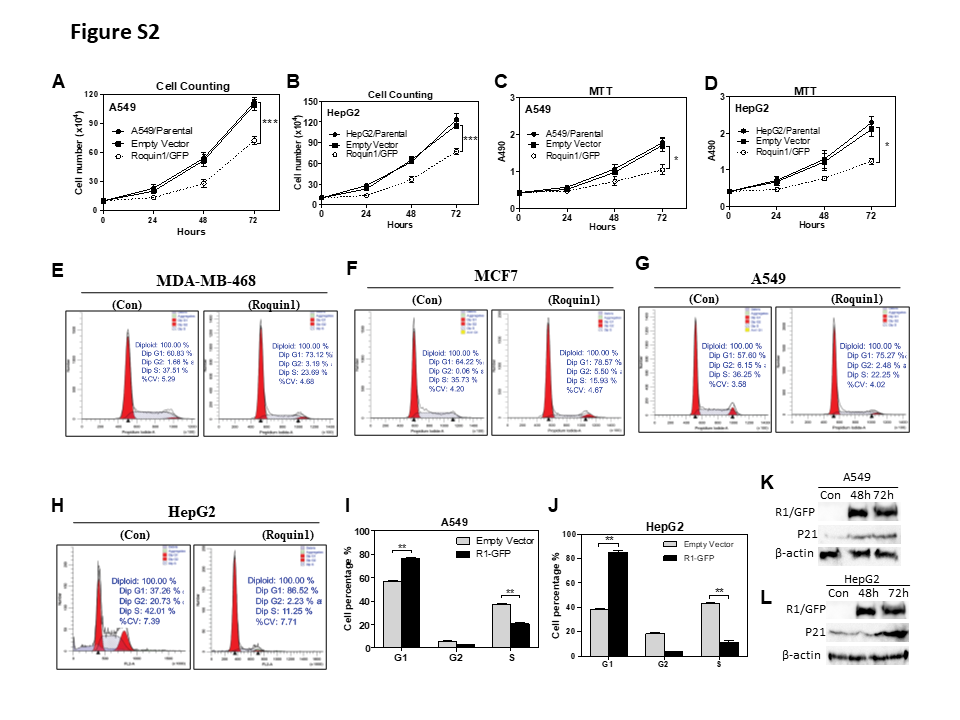


**Figure S2.** Roquin1 inhibits cell proliferation and induces G1/S phase cell cycle arrest in tumor cells. **a-b** Cell counting was carried out every 24 h in A549 (**a**) and HepG2 (**b**) cells with Roquin1/GFP overexpression. ****P* < 0.001. **c-d** MTT assay was performed in A549 and HepG2 cells to measure cell proliferation after Roquin1 overexpression. **P* < 0.05. **e-h** Representative cell cycle histograms showing cell cycle analyses of MDA-MB-468 (**e**), MCF7 (**f**), A549 (**g**), and HepG2 (**h**) cells after Roquin1 overexpression. **i-j** Cell cycle analysis was carried out in A549 (**i**) and HepG2 (**j**) cells after Roquin1 overexpression, and the percentages of different cell phases were quantified. ***P* < 0.01. **k-l** The protein level of cell cycle inhibitor p21 was measured by immunoblotting with an anti-p21 antibody at different time points after Roquin1 overexpression in A549 (**k**) and HepG2 (**l**) cells. β-actin was used as a loading control.


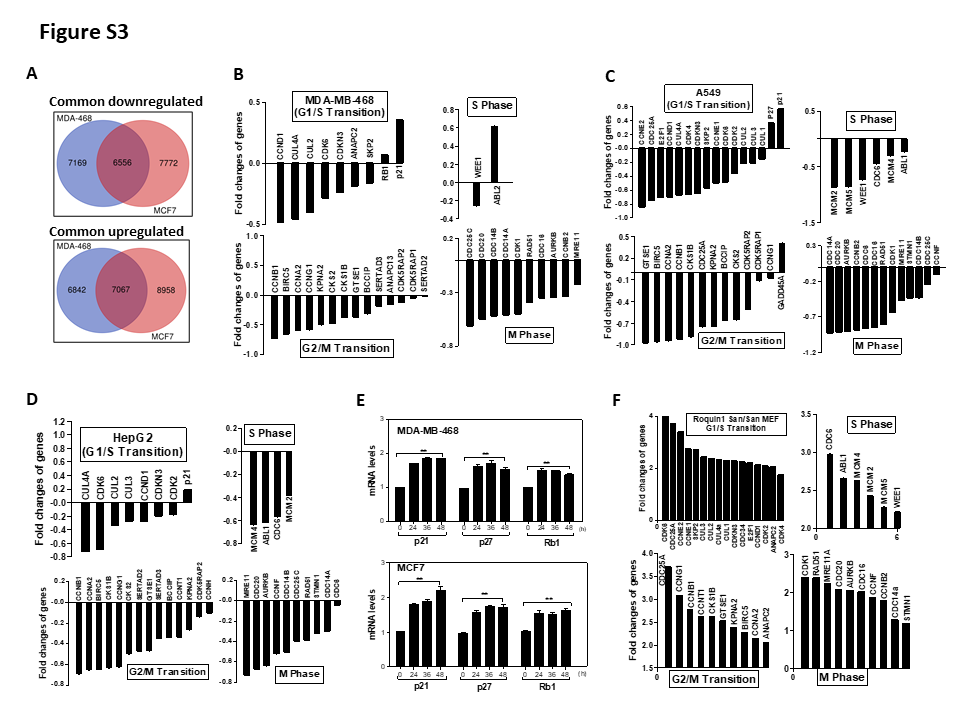


**Figure S3.** Roquin1 selectively inhibits the mRNA expression of cell cycle-promoting genes. **a** Venn diagrams showing the common down-regulated (upper) and up-regulated genes (bottom) by Roquin1 in breast tumor cells. **b-d** The expression levels of cell cycle–related genes affected by Roquin1 were analyzed by RNA-seq in MDA-MB-468 (**b**), A549 (**c**), and HepG2 (**d**) cells. The expression of genes promoting G1/S, S phase, G2/M, and M phase transition was downregulated; and the expression of cell cycle–inhibiting genes were upregulated. **e** The mRNA expression levels of indicated cell cycle–inhibiting genes were measured by qPCR in MDA-MB-468 and MCF7 cells at different time points, including *p21*, *p27*, *p16*, and *Rb1*. **f** Cell cycle–related genes were regulated by Roquin1 in Roquin1^san/san^ MEF cells. The expression of genes promoting G1/S, S phase, G2/M, and M phase transition were upregulated.


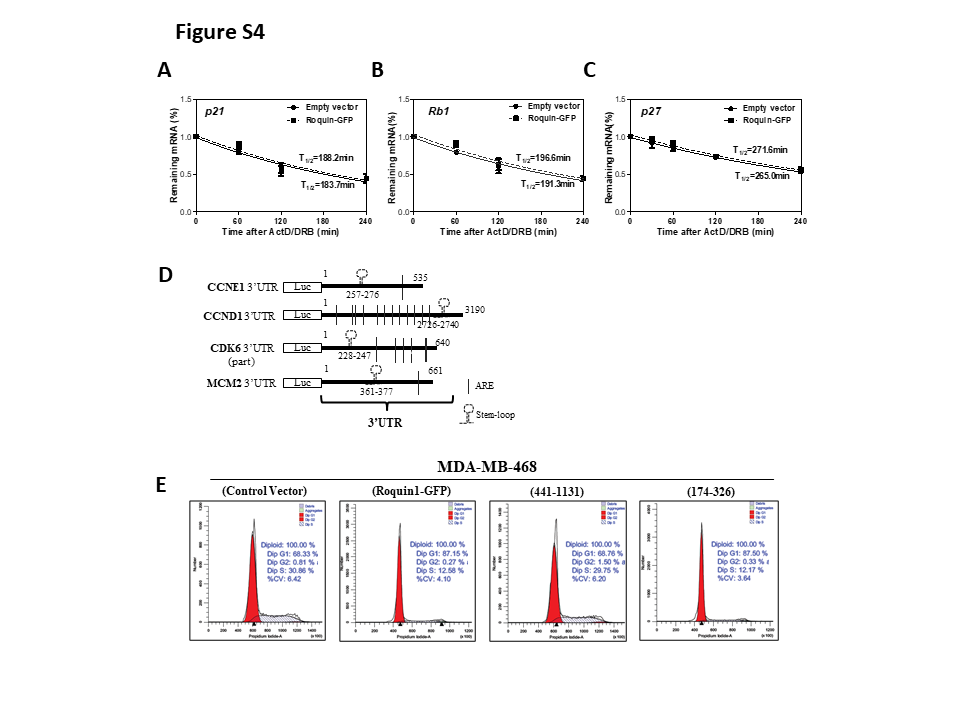


**Figure S4.** Roquin1 destabilizes the mRNAs of cell cycle-promoting genes via the ROQ domain. **a**–c The half-lives of cell cycle–inhibiting genes, including *p21* (**a**)*, p16* (**b**)*,* and *p27* (**c**) were measured by qPCR in Roquin1-expressing MDA-MB-468 cells. **d** Schematic representation of the luciferase reporter constructs containing 3’UTRs sequences of *CCNE1*, *CCND1*, *CDK6* (part), and *MCM2*. **e** Representative cell cycle histograms showing cell cycle analyses of MDA-MB-468 cells after overexpression of Roquin1/GFP, aa 441-1131, and aa 174-326 truncated mutations.


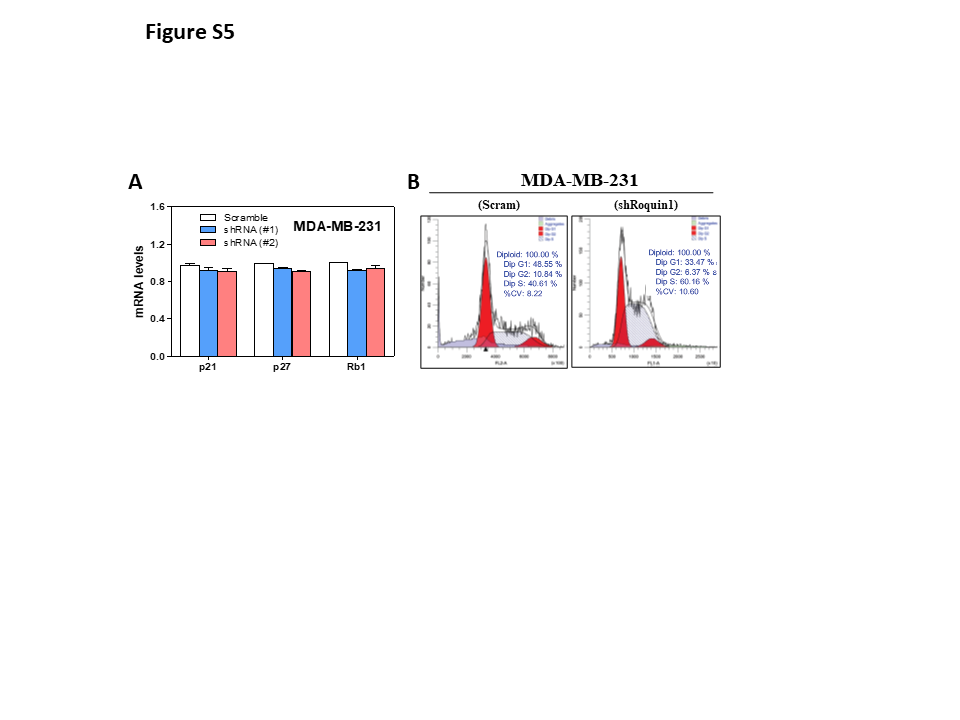


**Figure S5.** Knocking down Roquin1 enhances breast tumor cell cycle progression. **a** The mRNA expression levels of indicated cell cycle–inhibiting genes were measured after Roquin1 knockdown by infecting lentivirus expressing shRNA/Scramble and shRNA/Roquin1 by qPCR in MDA-MB-231 cells*.* **b** Representative cell cycle histograms showing cell cycle analyses of MDA-MB-231 cells after knocking down Roquin1.


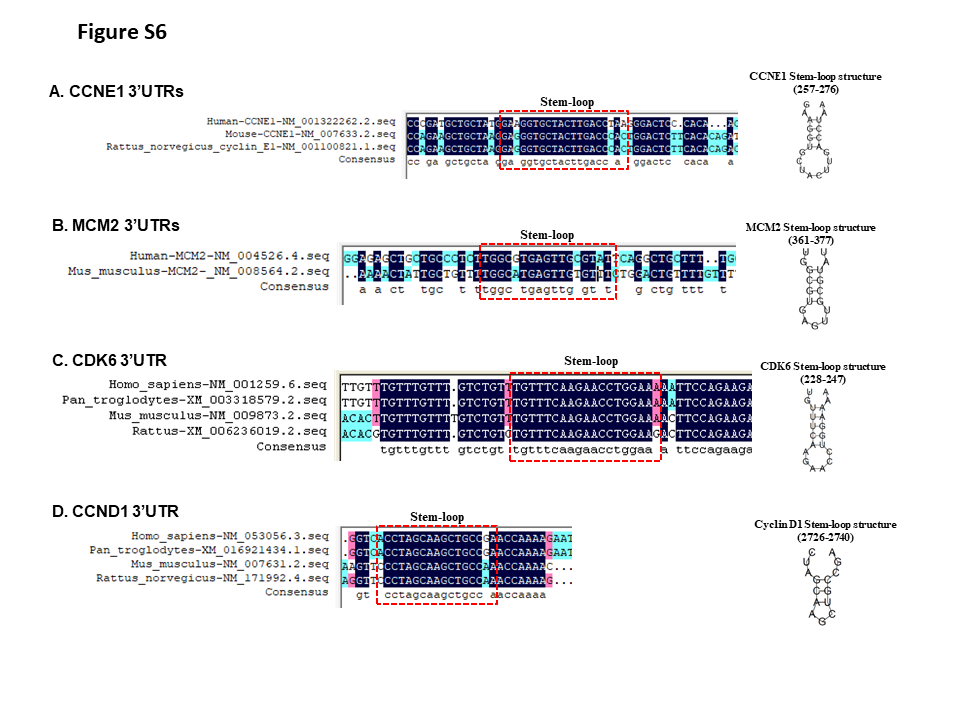


**Figure S6.** Putative stem-loop structure in the 3’UTRs of cell cycle-promoting genes. **a**–**d** The 3’UTR sequences from different species for each cell cycle–promoting gene, including *CCNE1* (**a**), *MCM2* (**b**), *CDK6* (**c**), and *CCND1* (**d**), was aligned using DNAMAN software. The stem-loop sequences were predicted by RNAfold web server to fold a secondary stem–loop structure (right) and indicated by red box.


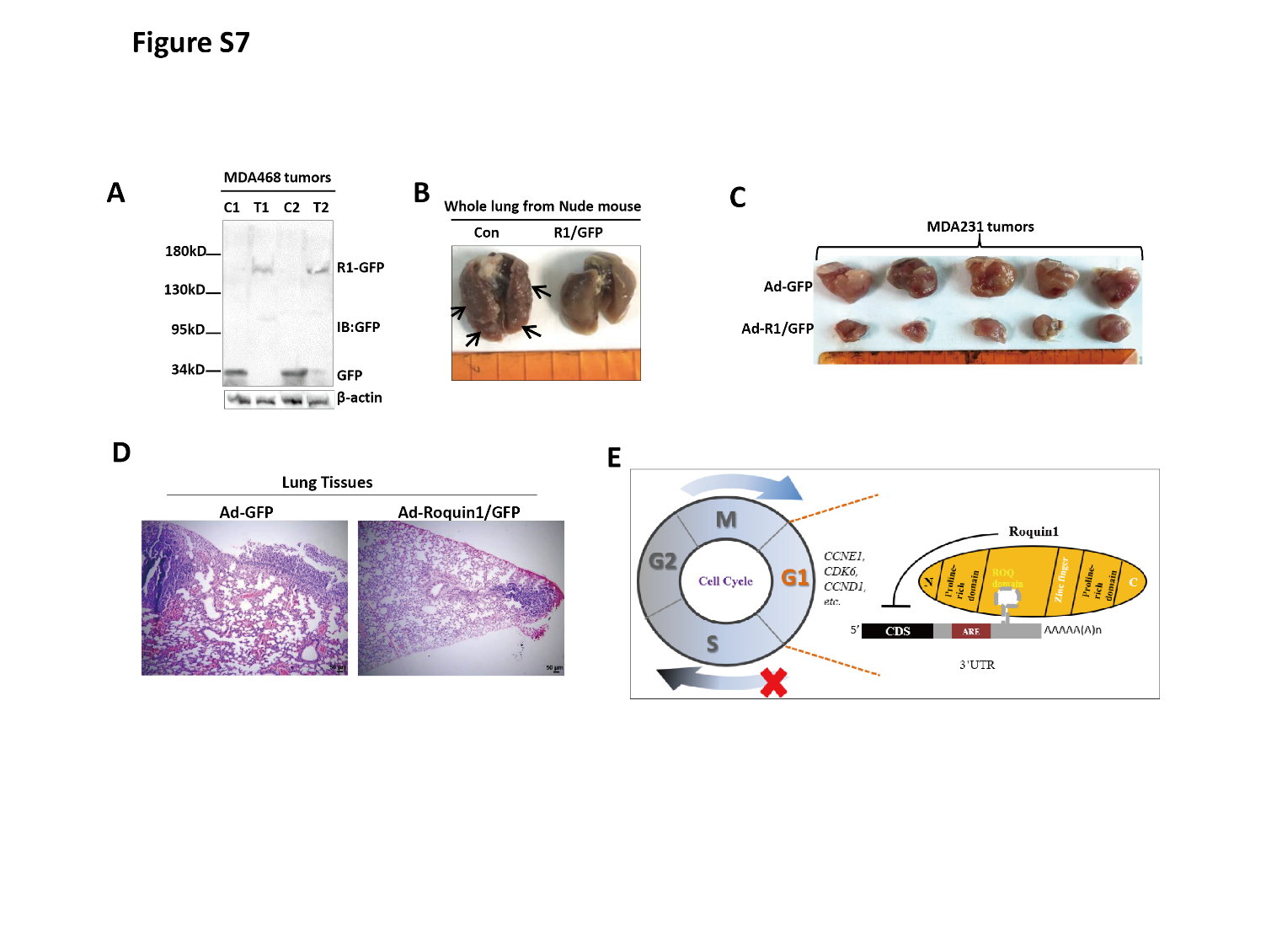


**Figure S7.** Roquin1 suppresses breast tumor growth and metastasis. **a** Total protein was extracted from tumor tissues and used to detect Roquin1/GFP expression by immunoblotting with an anti-GFP antibody. **b** Whole lungs from nude mouse bearing MDA-MB-468/GFP or MDA-MB-468/Roquin1/GFP tumors was collected and compared. **c** MDA-MB-231 tumors treated with control adenovirus (Ad-GFP) or Roquin1-expressing adenovirus (Ad-R1/GFP). **d** H&E staining of lung sections of tumor-bearing mice treated with control adenovirus or Roquin1-expressing adenovirus. Scale bar, 50µm. **e** A proposed work model of cell cycle-promoting genes regulation by Roquin1.
