# Supplementary material for: Roquin1 inhibits the proliferation of breast cancer cells by inducing G1/S cell cycle arrest via selectively destabilizing the mRNAs of cell cycle–promoting genes
Source: J Exp Clin Cancer Res. 2020 Nov 23;39:255. doi: 10.1186/s13046-020-01766-w (PMC7686734; doi:10.1186/s13046-020-01766-w)
Supplement: Supplementary file 2 — Additional file 2. [file 13046_2020_1766_MOESM2_ESM.docx]

**Additional file 2: Supplementary Materials and Methods**

***Tumor tissue samples***

19 human breast tumor samples and their matched surrounding ‘healthy’ tissues were obtained between October 2018 and October 2020 from Beijing Jishuitan Hospatial, the 4^th^ Clinical Medical College of Peking University. The tumors have mixture molecular subtypes, including eight luminal A, three luminal B, and five triple negative breast tumors. The subtypes of the rest three tumors are unknown. All subjects gave their informed consent for inclusion before they participated in the study. The experimental protocols were approved by the Ethics Committee of Beijing Jishuitan Hospatial, the 4^th^ Clinical Medical College of Peking University and the Medical Ethics Committee of the Institute of Microcirculation, CAMS & PUMC.

***RNA isolation and qRT-PCR***

Total RNA was extracted with TRIzol from tissues or cultured cells and reverse transcribed to cDNA for qPCR using SYBR green Fast Master Mix (Roche). Gene expression level was based on the ∆∆Ct method and normalized to GAPDH.

***Antibodies and Reagents***

Polyclonal rabbit anti-Roquin1 (ab70195), anti-GFP (ab290), anti-p21 (ab227443), and normal rabbit IgG (ab37415) were from Abcam. Polyclonal rabbit anti-PARP1 (9542S) and anti-caspase3 (96625) antibodies were from Cell Signaling Technology. Rabbit anti-CCNE1 (11554-1-AP), anti-MCM2 (10513-1-AP) and mouse anti-GAPDH (60004-1-Ig) antibodies were from Proteintech Co. Monoclonal β-actin (A2066) antibody was from Sigma Company. Thiazolyl Blue Tetrazolium Bromide (MTT) (M2128), actinomycin D (ActD, A1410), and 5, 6-Dichlorobenzimidazole 1-β-D-ribofuranoside (DRB, D1916) were from Sigma-Aldrich (St. Louis, USA). G418 (G8168) and puromycin (P8833) were also from Sigma. Protein A/G PLUS-Agarose beads were from Sant Cruz Biotechnology. GFP-coated beads (GFP-Trap Dynabeads, GTD20) were from ChromoTek (Germany). Dynabeads M-280 streptavidin (60210) were from Invitrogen.

***Western Blotting***

Cell or tissue samples were collected and lysed with a modified RIPA buffer containing PMSF and protease inhibitor cocktail (Roche). Protein concentration was measured by BCA method. Equal amount of protein lysates was subjected to electrophoresis by SDS-PAGE and transferred onto a polyvinylidene membrane. The membrane was then blocked with 5% fat-free milk and incubated with primary antibody (dilution: 1:1000 in 5% fat-free milk) overnight at 4℃. The band was detected with HRP-conjugated secondary antibodies (dilution: 1:5000 in 5% fat-free milk) using ECL chemiluminescent detection method.

***Cell counting assay***

Cell counting assays were conducted in 6-well plates. Cells were plated at the total number of 10 x 10^4^ cells per well and cultured with DMEM or RPMI-1640 with 10% FBS plus 1% Peni/Stro. Cells were counted after 24, 48, and 72 hrs. Each cell line was plated in triplicate and the experiment was repeated at least three times with consistency.

***MTT assay***

10^4^ cells per well were cultured in 96-well plate with DMEM or RPMI-1640 with 10% FBS plus 1% Peni/Stro. A total of 20μL of MTT dilution in PBS was added per well and cells were incubated for 90 mins. Absorbance was measured at 570 nm using a Bio-Tek Elx800 Series Universal Microplate Reader.

***Apoptosis analysis***

MDA-MB-468 and MCF7 cells overexpressed Roquin1/GFP and GFP were cultured for 48h. 1×10^6^ cells were harvested and washed twice with PBS, then stained with PE-conjugated Annexin V and 7-AAD (559763, BD, USA) for 15 min at room temperature in dark. Cells were analyzed by flow cytometry (NovoExpress 1.4.1).

***RNA affinity binding assay***

150 pmols biotinylated RNA oligonucleotide probes with stem-loop structure (wt) or without (mut1) were bound to 100 µL of streptavidin-coated magnetic beads (Dynabeads M-280, Invitrogen) for 30 min at room temperature with gentle rotation on a rocker. The coated beads were then washed three times with washing buffer according to the company’s instruction. 10 mg total protein of MDA-MB-468/Roquin1 was then added and incubated for 2 h at 4℃. Beads were then washed six times in B&W Buffer (Invitrogen). The bound proteins were eluted with loading buffer, boiled, and separated by 10% SDS-PAGE, followed by immunoblotting analysis with anti-GFP antibody.

***RIP-ChIP***

Roquin1/GFP fusion protein was expressed in MDA-MB-468/Roquin1 cells for 30 h. Then, cells were cross-linked for 10 min by addition of formaldehyde (1% v/v). Glycine was used to stop crosslinking (125 mM). Cells were washed with cold PBS, resuspended in 500 μL of polysome lysis buffer, and placed on ice for 5 min. Cell lysates were collected by centrifugation at 10,000 g for 10 min at 4℃, and re-suspended in 500 μL of polysome lysis buffer. Then, the lysates were sonicated and pre-clear with rabbit IgG to remove non-specific background. Pre-cleared lysates were used for IP with GFP-coated beads or rabbit IgG-coated beads at 4℃ for 4 h. After pull-down, 100 μL supernatants were taken out for Input. Each immune complex was washed five times with ice-cold NT2 buffer. RNA was isolated with Trizol reagent, and re-suspended in 50 μL of RNase-free water, followed by DNase I treatment and further detection.

***Sequence Alignments and Stem-loop Structure Prediction***

For 3’UTR stem-loop structure sequence conservation analysis of *Cyclin E1*, *MCM2*, *CDK6*, and *Cyclin D1*, the 3’UTR sequences were extracted for different species from the National Center for Biotechnology Information (NCBI) database: *Cyclin E1* 3’UTR: human (*Homo sapiens*; accession number NM_001322262.2), mouse (*Mus musculus*; NM_007633.2), rat (*Rattus*; NM_001100821.1); *MCM2* 3’UTR: human (*Homo sapiens*; NM_004526.4), mouse (*Mus musculus*; NM_008564.2); *CDK6* 3’UTR: human (*Homo sapiens*; NM_001259.6), mouse (*Mus musculus*; NM_009873.2), chimpanzee (*Pan troglodytes*; XM_003318579.2), rat (*Rattus*; XM_006236019.2); *Cyclin D1* 3’UTR: human (*Homo sapiens*; XM_053056.3), mouse (*Mus musculus*; NM_007631.2), chimpanzee (*Pan paniscus*; XM_016921434.1), rat (*Rattus*; NM_171992.4). Stem-loop sequence conservation analysis was performed using DNAMAN software. The stem-loop structure was predicted through RNAfold web server ( <http://rna.tbi.univie.ac.at/> ).
