# Supplementary material for: Roquin1 inhibits the proliferation of breast cancer cells by inducing G1/S cell cycle arrest via selectively destabilizing the mRNAs of cell cycle–promoting genes
Source: J Exp Clin Cancer Res. 2020 Nov 23;39:255. doi: 10.1186/s13046-020-01766-w (PMC7686734; doi:10.1186/s13046-020-01766-w)
Supplement: Supplementary file 5 — Additional file 5: Supplementary Table S3. List of primer and RNA-EMSA probes sequences used in this study. [file 13046_2020_1766_MOESM5_ESM.docx]

**Supplementary Table S1. List of primer and RNA-EMSA probes sequences used in this study.**

| Gene | Sense (5’-3’) | Anti-sense (5’-3’) |
| --- | --- | --- |
| *Roquin1* (qPCR) | AGAGAAGAGCAGCAGAAACCAGTGAT | CCCTGAGAAGGTATGTTTTGATGAGGTG |
| *GAPDH* (qPCR) | ACCTGCCAAATATGATGACATCAAGAAGGT | GTTGCTGTAGCCAAATTCGTTGTCATACC |
| *CCND1* (qPCR) | CCCTCGGTGTCCTACTTCAAA | CACCTCCTCCTCCTCCTCTTC |
| *CCNE1* (qPCR) | CCATCATGCCGAGGGAGC | GGTCACGTTTGCCTTCCTCT |
| *CDK6* (qPCR) | CCAGATGGCTCTAACCTCAGT | AACTTCCACGAAAAAGAGGCTT |
| *MCM2* (qPCR) | TCAGCATGGTCAAGTACAACTGCAACAA | TGTTGTGATAGATGCCAGTCAGCTCTATCT |
| *p21* (qPCR) | GGAGACTCTCAGGGTCGAAA | GGATTAGGGCTTCCTCTTGG |
| *p27* (qPCR) | AGTGTCTAACGGGAGCCCTA | CCGGGTTAACTCTTCGTGGT |
| *Rb1* (qPCR) | CAGGCTTGAGTTTGAAGAAACAGAAGAAC | ATCTCATCTAGGTCAACTGCTGCAATAAAG |
| *TNF* (qPCR) | TCTCCTACCAGACCAAGGTCAA | CAGACTCGGCAAAGTCGAGATAGT |
| *CCNE1* (full-length 3UTR) | T**TCTAGA**CCACCCCATCCTTCTCCACCAAAGACAG | **GGCCGGCC**TTTCAAAAACAGTATTATCTTTATTAAA |
| *CCND1* (full-length 3UTR) | T**TCTAGA**GGGCGCCAGGCAGGCGGGCGCCACCGCCAC | **GGCCGGCC**AACATGTAACCGGCATGTTTCCAGCAGAA |
| *MCM2* (full-length 3UTR) | T**TCTAGA**GGCCCTATGCCATCCATAAGGATTCCTTGGGATTC | **GGCCGGCC**TTATTAAAAATTAAAACTACAGAA ACCAAACCG |
| *CDK6* (part 3UTR) | T**TCTAGA**GGCCTCAGCAGCCGCCTTAAGCTGATCCTGCG | **GGCCGGCC**AGCAAATTTACTGCTTTTGGCCAGAAAAGAAAT |
| *β-actin* (full-length 3UTR) | T**TCTAGA**GCGGACTATGACTTAGTTGCGTTACACCCTTTCTTGACAA | **GGCCGGCC**TTTAAGGTGTGCACTTTTATTCAACTGGTCTCAAG |
| *p21* (full-length 3UTR) | T**TCTAGA**TCCGCCCACAGGAAGCCTGCAGTCCTGGAA | **GGCCGGCC**TAAAGTCACTAAGAATCATTTATTGAGCACCT |
| *β-actin* (w/CCNE1 stem-loop) | GAAGGTGCTACTTGACCTAAACTGACTTGAGACCAGTTGAAT | GTACAGGTAAGCCCTGGCTGCCT |
| *β-actin* (w/MCM2 stem-loop) | TGGCGTGAGTTGCGTATACTGACTTGAGACCAGTTGAAT | GTACAGGTAAGCCCTGGCTGCCT |
| *CCNE1* (RNA-ChIP) | CAGTGCGTGCTCCCGATGCTGCTAT | CGTGGCCCTCCACAGCTTCAAGCTTTT |
| *MCM2* (RNA-ChIP) | TTCTTACATGGATGTCAGGAGAGCTG | GATTACAAACATCTTCAACCAGCTCTC |
| *CCNE1* (truncated 3UTR) | T**TCTAGA**CCACCCCATCCTTCTCCACCAAAGACAG | **GGCCGGCC**CATAGCAGCATCGGGAGCACGCACT |
| *MCM2* (truncated 3UTR) | T**TCTAGA**GGCCCTATGCCATCCATAAGGATTCCTTGGGATTC | **GGCCGGCC**AGAGGGCAGCAGCTCTCCTGA CATCCAT |
| *CCNE1* (3UTR-mut1) | TGCTATGGAA**T**GTGCTACTTGAC**T**TAAGGGACTCC | GCATCGGGAGCACGCACTGGTGTCTGGAGGTGGCT |
| *CCNE1* (3UTR-mut2) | TGCTATGGAA**CC**TGCTACTTGA**GG**TAAGGGACTCC | GCATCGGGAGCACGCACTGGTGTCTGGAGGTGGCT |
| *MCM2* (3UTR-mut1) | TGCCCTCTTGG**AA**TGAGTTG**TT**TATTCAGGCT | GCAGCTCTCCTGACATCCATGTAAGAAGGCTAACAC |
| *MCM2* (3UTR-mut2) | TGCCCTCTTGG**GC**TGAGTTG**GC**TATTCAGGCT | GCAGCTCTCCTGACATCCATGTAAGAAGGCTAACAC |
| *CCNE1* (WT-Probe): 5’-GAAGGUGCUACUUGACCUAA-3’  *CCNE1* (mut1-Probe): 5’- GAA**U**GUGCUACUUGAC**U**UAA-3’  *CCNE1* (mut2-Probe): 5’-GAA**CC**UGCUACUUGA**GG**UAA-3’ | | |
| *MCM2* (WT-Probe): 5’-UGGCGUGAGUUGCGUAU-3’  *MCM2* (mut1-Probe): 5’-UGG**AA**UGAGUUG**UU**UAU-3’  *MCM2* (mut2-Probe): 5’-UGG**GC**UGAGUUG**GC**UAU-3’ | | |
